# Germ Diseases

**Published:** 1893-01

**Authors:** 


### GERM DISEASES.

Of all the agencies in the spread of what are called " germ diseases," dust is, perhaps the most potent. The peculiar dangers of the month of March are due not merely to the high winds then prevalent, but to the dust with which the wind pollutes the air at that season. Inorganic dust—that is, dust composed of nothing but pulverized earth—is not of itself very harmful. Thus coal miners are not especially prone to lung diseases, though working in coal dust constantly. Dust, however, as found in the streets of our cities and towns, is largely made up of organic matter. Such dust, if not of itself largely composed of germs, still furnishes an excellent lurking place for them. Not all germs are harmful or generative of inflammation ; but, on the other hand, some of the germs most harmful to human life are constantly to be found where street dust is abundant. When we inhale dust, therefore, we undoubtedly inhale many germs, or bacteria, which may or may not find a lodging place within us. It is almost needless to say that the mucous membrane of the nose is a much safer place for the reception of dust than that of the lungs, and we should therefore keep the mouth closed when we are forced to inhale dust.

Long dresses that sweep the street in walking are fearful agents in the acquisition of disease, and it would not be a great stretch of authority for our Boards of Health to forbid their use as endangering the public health.

